# Spatial–Temporal Distribution of Fish Larvae in the Pearl River Estuary Based on Habitat Suitability Index Model

**DOI:** 10.3390/biology12040603

**Published:** 2023-04-15

**Authors:** Dongliang Wang, Jing Yu, Zhaojin Lin, Pimao Chen

**Affiliations:** 1 Guangdong Provincial Key Laboratory of Fishery Ecology and Environment China, Scientific Observing and Experimental Station of South China Sea Fishery Resources & Environment, Ministry of Agriculture and Rural Affairs, South China Sea Fisheries Research Institute, Chinese Academy of Fishery Sciences, Guangzhou 510300, China; 2 State Key Laboratory of Tropical Oceanography, South China Sea Institute of Oceanology, Chinese Academy of Sciences, Guangzhou 510301, China; 3 University of Chinese Academy of Sciences, Beijing 100049, China

**Keywords:** spawning ground, environmental effects, remote sensing, suitability assessment

## Abstract

**Simple Summary:**

The Pearl River Estuary (PRE), China, where riverine and marine factors alternate in strength and weakness, is one of the most important fishing grounds in the South China Sea. The spatial–temporal distribution of fish larvae directly affects the stock density in the area. This study revealed the relationship between fish larvae and marine environmental factors in the PRE, through multisensor satellite remote sensing and fishing surveys, based on the Habitat Suitability Index (HSI) model. The spatial–temporal distribution of fish larvae has unique distribution characteristics under different seasons and has high consistency with the changes of monsoon and flow field, etc. The findings of this study can help researchers gain a good understanding of the movement of fish larvae in the PRE and promote better habitat conservation under a changing climate.

**Abstract:**

The spawning grounds are important areas for the survival and reproduction of aquatic organisms and play an important role in the replenishment of fishery resources. The density of fish larvae in the Pearl River Estuary (PRE) was analyzed to establish Habitat Suitability Index (HSI) based on marine environmental factors. Survey data and satellite remote sensing data, including sea surface temperature, sea surface salinity and chlorophyll a concentration, from 2014 to 2017 during April–September were analyzed. Results showed that the accuracy of the HSI model based on the larval density and environmental factors was more than 60%, and the distribution trend of HSI was consistent with the distribution trend of larval density. The HSI models constructed based on Arithmetic Mean Model (AMM), Geometric Mean Model (GMM) and Minimum Model (MINM) methods can better predict the spatial–temporal distribution of larvae in the PRE. Among them, the accuracy of the HSI model constructed by the AMM and GMM methods was the highest in April (71%) and September (93%); the accuracy of the HSI model constructed by the MINM method was the highest in June (70%), July (84%) and August (64%). In general, the areas with high HSI values are mainly distributed in the offshore waters of the PRE. The spatial–temporal distribution of larvae in the PRE was influenced by monsoon, Pearl River runoff, Guangdong coastal currents and the invasion of high-salinity seawater from the outer sea.

## 1. Introduction

The Pearl River Estuary (PRE) is a subtropical, high productive estuary in the northern South China Sea (SCS). The Pearl River is the largest river in southern China. It is an important fishery production base and a gene bank of aquatic biological resources, with more than 380 species of fish [1]. The runoff from the Pearl River flows into the SCS through eight main entrances, providing an estuary habitat with a unique natural environment and rich species composition [2,3]. The PRE is located at the junction of salty and fresh water and is driven by runoff, monsoons and shelf circulation. In the south of it, there are the Wanshan Islands, with more than 70 islands, which are densely distributed, forming a unique island chain structure and complex flow field. Local upwelling and dilute water masses can bring a large amount of deposited nutrients into the water body, resulting in high primary productivity and rich bait organisms [4,5,6], which is a major area for spawning, baiting, breeding and migration of fishes [7,8,9]. From 2013 to 2016, 285 species of fish were found in gillnet and ground cage surveys in the PRE [10,11]. In the area, fishery resources have been depleted due to economically important and overfishing, with egg and larvae species halved, compared to the 1990s. The dominant species have changed to low-value species, and the proportion of economic species is low [7,12,13]. In the marine ecosystem, larvae are major predators and important consumers of secondary productivity [14]. The larvae development period is the most vulnerable stage and a very short period in the fish life cycle, as well as a transitional period with significant changes in morphological, physiological and ecological characteristics, whose survival rate and quantity are fundamental to the replenishment sustainable use of stock [15,16]. Investigation of larvae is an essential research in fish population dynamics and marine ecology. Research on the conservation of larvae determines the abundance of resources. Moreover, studies have shown that the spatial distribution of larvae is closely related to environmental factors, such as water temperature, salinity, chlorophyll concentration and flow field, in the sea area [17,18,19,20]. Therefore, there is an urgent need to study the living environment of the larvae in the PRE.

The Habitat Suitability Index (HSI) can be used to simulate the response of organisms to environmental conditions. The model has the advantages of a straightforward evaluation approach, clear expression of species’ optimal environmental circumstances and being easy to understand [21,22]. It is commonly utilized in the assessment of fishery resources and the forecast of central fishing grounds [23,24,25,26]. HSI model was used to assess the effects of temperature and dissolved oxygen on the distribution of *Thunnus obesus* in the Indian Ocean [27], the relationship between fishing grounds of *Sthenoteuthis oualaniensis* and sea surface temperature, sea surface height anomalies in the SCS [28] and the relationship between *Larimichthys polyactis* and environmental factors in Haizhou Bay [29]. Previous studies focused on the biological characteristics and population variations of larvae [16,30,31]. The response mechanisms between larvae and habitat environmental variables are unclear.

In this study, the spatial and temporal distribution of larvae in the PRE was analyzed based on the HSI model, and the effects of environmental changes on the distribution of larvae were analyzed to predict the location and abundance of larvae in the PRE. The findings of the study will help to clarify the status of fishery resources replenishment in the area and provide scientific reference for the restoration and conservation of offshore spawning grounds in the SCS.

## 2. Materials and Methods

### 2.1. Fishery Data

**Figure 1:**
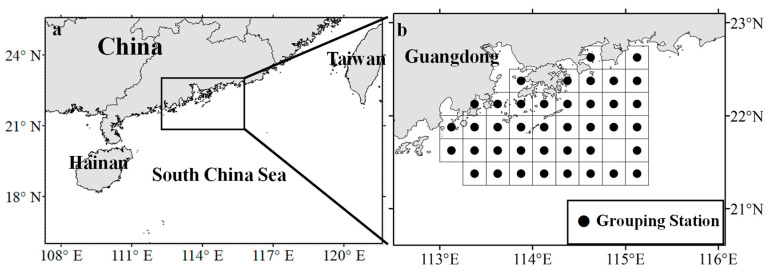
(**a**) Research area; (**b**) grouping stations.

The larval data were obtained from surveys of spawning grounds in the PRE conducted between 2014 and 2018 (April, June, July, August and September). The research area was at 113–115° E, 21–23° N (Figure 1). The sampling method was implemented in accordance with Specifications for oceanographic survey—Part 6: Marine biological survey (GB/T 12763.6-2007). The plankton nets (shallow water type I) were used in the waters less than 30 m, and macroplankton nets were used in the waters more than 30 m. The trawling depth from the bottom to the surface and different water layers were not sampled separately. In the area of water depth less than 50 m, sampling every 3 h, samplings were performed a total of 9 times; in the area of water depth greater than 50 m, sampling every 4 h, samplings were performed a total of 7 times. During the trawling, the wire rope inclination angle was less than 45° [32]. The larvae were identified by morphological characteristics. In this study, larval data were grouped by 0.25° × 0.25° grid cells, and the formula for calculating the density of individual larvae is as follows.

(1) $$C=\frac{N}{V}$$

where C is the larval density, in ind(10 m)^−3^; *N* is the number of larvae, in ind and *V* is the volume of filtered water, in (10 m)^−3^.

### 2.2. Satellite Remote Sensing Data

Satellite data include sea surface temperature (SST), sea surface salinity (SSS) and sea surface chlorophyll a concentration (Chl-a). SST and Chl-a data were obtained from MODIS Aqua products (http://oceancolor.gsfc.nasa.gov; accessed on: 20 May 2021) with a temporal resolution of 1 day and a spatial resolution of 4 km. SSS data were from the Global Ocean Physical Reanalysis Product of the Copernicus Marine Environment Management Service (CMEMS; http://marine.copernicus.eu/; accessed date: 20 May 2021) with a temporal resolution of 1 month and a spatial resolution of 1/12°. R v.4.0.0 (R Development Core Team, 2020) software was used to eliminate invalid values, month-by-month average, and data fuse for SST, SSS and Chl-a. ArcGIS 10.3 (Esri, Redlands, CA, USA) was used to map the geographically overlaid distribution of environmental parameters and larval density [33,34,35]. The “stats” package in software R v.4.0.0 was used to construct and test the HSI model.

### 2.3. Suitability Index Model

The Suitability Index (SI) models were established using the larval density with SST, SSS and Chl-a, respectively. In this study, the area with the highest density of larvae is assumed to be the most suitable area for larvae, with an HSI of 1. The area with no larval density is unsuitable for larvae, with an HSI of 0. Among them, 80% of the larval density data were used to develop the model via random sampling, and the remaining 20% of the data were used for model validation. The single-factor habitat index SI was calculated as [36]:

(2) $${\mathrm{SI}}_{i}=\frac{Y_{i}}{Y_{i,\max}}$$

where ${\mathrm{SI}}_{i}$ is the suitability index for month i; $Y_{i}$ is the larval density for month i and $Y_{i,\max}$ is the maximum larval density caught in month i.

### 2.4. Habitat Suitability Index Model

The relationship between SI and SST and SSS and Chl-a was modeled by the simple nonlinear regression [37]. The equation is given as follows:

(3) $${\mathrm{SI}=e}^{A\times {\left(B+X\right)}^{2}}$$

where A and B are the coefficients in the model, and X is the value of each environmental factor.

HSI was calculated based on the single-factor SI using Maximum Model (MAXM), Minimum Model (MINM), Arithmetic Mean Model (AMM) and Geometric Mean Model (GMM), respectively, with the following equations [38,39]:

(4) $${\mathrm{HSI}}_{\mathrm{MAXM}\;}=\mathrm{MAX}\left[\left({\mathrm{SI}}_{\mathrm{SST}}\right),\left({\mathrm{SI}}_{\mathrm{SSS}}\right){,(\mathrm{SI}}_{\mathrm{Chl}-a})\right]$$

(5) $${\mathrm{HSI}}_{\mathrm{MINM}}=\mathrm{MIN}\left[\left({\mathrm{SI}}_{\mathrm{SST}}\right),\left({\mathrm{SI}}_{\mathrm{SSS}}\right){,(\mathrm{SI}}_{\mathrm{Chl}-a})\right]$$

(6) $${\mathrm{HSI}}_{\mathrm{AMM}}=[\left({\mathrm{SI}}_{\mathrm{SST}}\right)+\left({\mathrm{SI}}_{\mathrm{SSS}}\right)+\left({\mathrm{SI}}_{\mathrm{Chl}-a}\right)]/4$$

(7) $${\mathrm{HSI}}_{\mathrm{GMM}\;}=\sqrt[{4}]{\left({\mathrm{SI}}_{\mathrm{SST}}\right)\;\times \;\left({\mathrm{SI}}_{\mathrm{SSS}}\right)\;\times \;\left({\mathrm{SI}}_{\mathrm{Chl}-a}\right))}$$

where ${\mathrm{SI}}_{\mathrm{SST}}$, ${\mathrm{SI}}_{\mathrm{SSS}}$ and ${\mathrm{SI}}_{\mathrm{Chl}-a}$ were the SI values corresponding to SST, SSS and Chl-a, respectively.

The remaining 20% of investigation data were taken into each of the 4 equations for validation. The forecast was considered accurate if the error between theoretical value and actual value was less than 0.4, otherwise, the forecast is considered inaccurate [40]. ArcGIS 10.3 software was used to map HSI distribution and validate the model simulation.

## 3. Results

### 3.1. SI of Environmental Factors

The SI for each environmental factor and larval density were obtained for different months (Table 1), based on the univariate linear regression. Significance tests (*p* < 0.05) indicated that each model was accurate.

### 3.2. SI Curve Distribution and Suitable Environment Range

**Figure 2:**
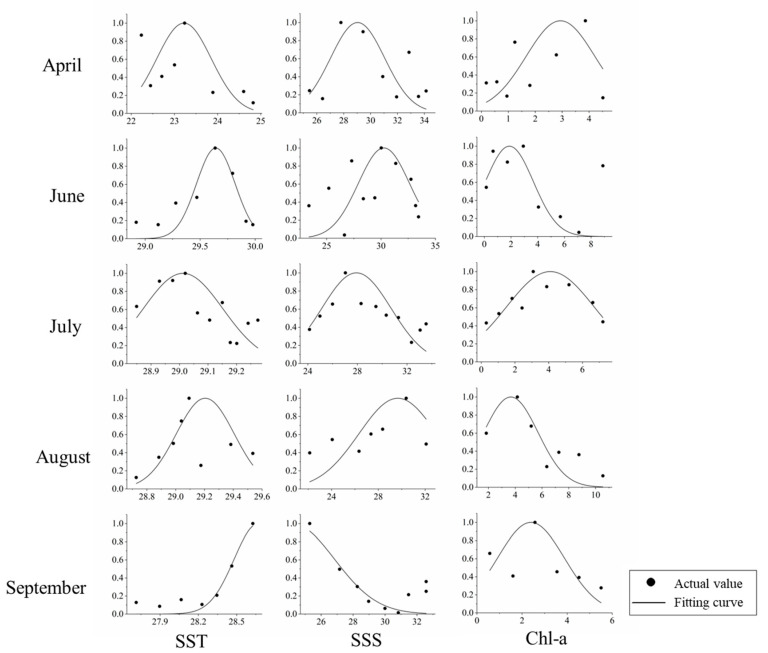
Comparison between actual larval density and fitting results in the PRE.

The three factors SST, SSS and Chl-a were fitted separately, and the actual values of larval density were plotted against the fitted results (Figure 2). Results of the SI models for the fitted factors for each month reflected the variation of larval density with environmental factors, with all models showing a single-peaked distribution trend and generally accurate simulation results. The first-order derivative of each SI model was made equal to zero, and the value of each factor at the highest SI was calculated to be the optimum value for larvae in that month (Table 2).

For SST, the optimum SST for April larvae was 23.2 °C, for June larvae was 29.6 °C, for July larvae was 29.0 °C, for August larvae was 29.2 °C and for September larvae was 28.7 °C (Figure 2, Table 2).

For SSS, the optimum SSS for April larvae was 29.0, for June larvae was 30.2, for July larvae was 27.9, for August larvae was 29.6 and for September larvae was 24.4 (Figure 2, Table 2).

For Chl-a, the optimum Chl-a for April larvae was 2.9 mg/m^3^, for June larvae was 1.9 mg/m^3^, for July larvae was 4.1 mg/m^3^, for August larvae was 3.7 mg/m^3^ and for September larvae was 2.4 mg/m^3^ (Figure 2, Table 2).

### 3.3. Validation of HSI Model Accuracy

The HSI model was established using the AMM, GMM, MAXM and MINM methods, respectively, and the data used to test the models were input into the models to obtain the theoretical HSI values, which were compared to the actual SI values. The model is considered correct if the difference between the HSI value and the actual value is less than 0.4 [40]. The accuracy validation of HSI models for each month was provided in Table 3.

In April, the AMM and GMM methods had the highest accuracy (71%), and the MAXM and MINM methods had the lowest accuracy (57%). In June, the MINM method had the highest accuracy rate (70%), and the MAXM method had the lowest accuracy rate (41%). In July, the MINM method had the highest accuracy rate (84%), and the MAXM method had the lowest accuracy rate (46%). In August, the MINM method had the highest accuracy (64%), and the AMM, GMM and MAXM methods had the lowest accuracy (36%). In September, the AMM and GMM methods had the highest accuracy (93%), and the MAXM method had the lowest accuracy (60%).

**Figure 3:**
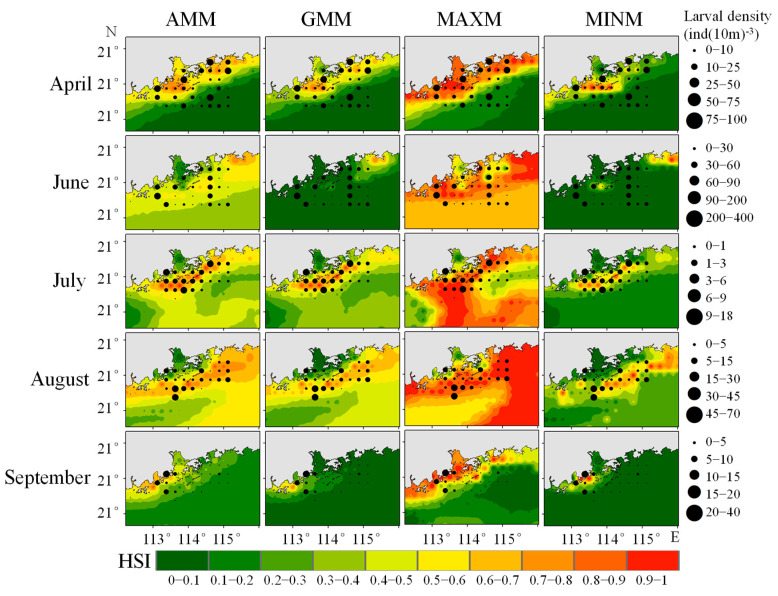
Spatial–temporal distribution of HSI predictions and actual larval density in the PRE.

The spatial–temporal distributions of HSI and larval density in the PRE indicated that the predicted results of the HSI model were similar to the actual larval density distribution trends (Figure 3). In April, the AMM and GMM methods had the highest forecast accuracy. In terms of spatial distribution, the nearshore area of the PRE (113–115° E, 21.5–22.5° N) had a higher density of larvae (>75 ind(10 m)^−3^) and a higher HSI (>0.8) predicted for this area based on the AMM and GMM methods. In the offshore waters of the PRE (113–114° E, 20.5–21.5° N; 114–115° E, 20.5–22° N), the larval density was low (0–10 ind(10 m)^−3^). The HSI in this area predicted by AMM and GMM was also low (<0.2), and the predicted results were consistent with the actual values. The results predicted by MAXM overestimated the low-value area of larval density, which was inconsistent with the actual situation. MINM did not identify the high-value area on the east side of the PRE, and the predicted results were slightly worse than those of AMM and GMM. In June, the MINM predictions were the most accurate, the spatial distribution predicted by AMM was more similar to the actual distribution of larval density, and both GMM and MINM predictions gave a conservative estimate of larval density, resulting in some high-value areas not being detected. MAXM accurately predicted the high-value area of larval density, but it also overestimated areas of low larval density as well, which was not consistent with the actual situation. In July, the spatial distributions predicted by AMM, GMM and MINM were similar and consistent with the actual larval density distribution. Higher-density areas (113–115° E, 21.7–22.5° N) had higher HSIs (>0.8) predicted by the three models, and lower-density areas had lower HSIs. In August, the predicted HSI distribution based on AMM, GMM and MINM was consistent with the actual distribution trends of larval density. Among them, the predicted results of AMM were more optimistic and those of MINM were more conservative. The predicted results of MAXM were too optimistic and did not match the actual situation. In September, predicted results of AMM, GMM, MAXM and MINM showed similar trends. The HSI results obtained from AMM, GMM and MINM for the near-shore waters on the west side of the PRE (112.7–113.5° E, 21.5–22° N) were higher (>0.8), and the density of larvae was relatively high (>20 ind(10 m)^−3^) at most survey stations in this area, and the predicted results were consistent with the actual values. However, the MAXM prediction made a higher estimate for the waters within the PRE, which was not consistent with the actual situation.

## 4. Discussion

### 4.1. HSI Model Selection

MAXM, MINM, AMM and GMM are widely used in the construction of the HSI model [22,39]. The fit of the HSI obtained by different methods to the actual density distribution depends on the modeling objectives and the structural error of the model, and there are significant differences in the output of the different methods [41]. This study showed that MINM indicated the smallest suitable area for larvae, AMM and GMM indicated a larger suitable area for larvae, and the predicted spatial distribution trends were generally consistent, and MAXM indicated the largest suitable area for larvae. The four models predicted trends similar to the actual situation, with the most appropriate models being AMM and GMM for April and September and MINM for June–August (Table 3). AMM and GMM are currently the most widely used algorithms in the fisheries HSI [39]. Previous studies have shown that the AMM was appropriate in cases where there are a high number of single-factor SI minimal value [29], and AMM estimates were more stable and less susceptible to SI extremes [39]. Therefore, it was widely used in resource estimation and fishery analysis [28,29,36]. MINM is limited by the minimum SI and the predictions are conservative. It is suitable for modeling areas with low larval density and is usually used for the establishment and evaluation of protected areas and for predicting the migration of central fishing grounds [39,42]. The high prediction accuracy of MINM in this study is mainly due to the overall low larval density in the PRE, and the existence of extreme values of larval density at a few stations, which ensures the overall prediction accuracy by ignoring the extreme values. The results of the MAXM simulation were less accurate. As MAXM adopts the maximum value of each SI factor to construct the model, which is influenced by the limitation of the maximum SI factor, and there are extreme points with large larval density in each month in this study. Therefore, MAXM will make overly optimistic estimates of the larval density in the PRE, resulting in the final prediction results deviating from the actual situation.

### 4.2. Analysis of SI Model Fitting

Water temperature plays a key role in fish maturation and egg development [17]. Seasonal fluctuations in water temperature affect the migratory, spawning and other behaviors of estuarine species [14]. For example, warm seawater temperature prior to spawning season can promote grouper gonad development, while at lower seawater temperatures, the gonads are not fully developed, resulting in relatively low numbers entering the spawning grounds that year [18]. The development time of cod eggs decreased exponentially from 18 days to 7 days with increasing temperature [43]. In April, larvae in the PRE mainly inhabited the waters with SST of 22–25 °C, and the most suitable habitat SST for larvae was 23.2 °C according to the SI model, and the density of larvae was 0–100 ind(10 m)^−3^. From June to September, larvae in the PRE were mainly distributed in the waters with SST of 28–30 °C, and the suitable SST for larvae was 28.7–29.6 °C according to the SI model, and the density of juvenile fish was 0–700 ind(10 m)^−3^ (Figure 3). High values of larval density in June–September were significantly higher than those in April. This may be due to the fact that higher seawater temperatures from June to September promote the development of fish gonads, resulting in increased spawning activity [44]. It also shortened the development time of fish eggs and speeded up the hatching of eggs to young, resulting in an increase in the larval density [45,46,47].

Salinity is considered to be the key factor controlling the distribution and composition of estuarine fish [14]. Due to the input of freshwater from the Pearl River flush, SSS in the PRE has strong spatial and temporal variability [3], which can affect the development and hatching of eggs as well as the survival of larvae [17]. The present study showed that larvae in the PRE inhabited in the waters with an SSS of 22–34, the most suitable habitat SSS for larvae in April, June, July and August was 28–30 based on the SI model, with a normal distribution trend. Moreover, the optimum SSS for larvae did not change significantly in different months, indicating that the suitable SSS values of larvae did not change significantly with the seasonal changes of SSS in the PRE. This might be due to the fact that too high or too low salinity prevented the exchange of substances between fertilized eggs and the surrounding medium, thus reducing the hatching rate [48]. In September, larvae inhabited in the waters with an SSS of 25–33 in the PRE. Based on the SI model, the most suitable SSS for larvae was 24.4. The suitable salinity for larvae was reduced by about 4–5% compared to other months, partly related to the variation of larvae habitat influenced by the change of the flow field in September in this sea area (Figure 3).

Chl-a reflects the amount of phytoplankton in the area and is the primary food supply for zooplankton and some marine creatures [49,50]. Therefore, chl-a can be one of the major reference indicators in the analysis of larvae habitat. Studies have shown that the spawning grounds and major fishing grounds of major commercial fishes in the northern SCS may vary with the distribution of chlorophyll concentration [51,52]. This study indicated that the larvae in the PRE inhabited in the waters with Chl-a of 0–10 mg/m^3^, and the optimum Chl-a for larvae in April, June, July, August and September was 2–4 mg/m^3^ based on the SI model. The higher Chl-a area did not result in a high distribution of larva density (Figure 2), partly due to a lag in the influence of Chl-a, which was one of the important indicators of marine primary productivity in fishery resources [53]. In addition, the decrease in Chl-a in this area increased the transparency of seawater, which had a positive impact on the spawning activity of fish and contributed to the increase in the number of larvae [54].

### 4.3. Analysis of HSI Model Fitting

The ecological environment of the PRE is complex and variable. Studies showed that the monsoons in the PRE played the most important role in the surface shape and extension of the plume [55,56]. The seasonal variability of the freshwater expansion of the PRE flush is well correlated with the monsoon-driven shelf circulation [57]. The Pearl River plume interacts with and regulates Guangdong Coastal Currents (GCC) as it travels in the northern SCS [58]. In April, the Pearl River estuary is in the transition period from the northeast monsoon to the southwest monsoon, and freshwater from the Pearl River flushes mainly in a westerly direction [4]. At this time, larval density is higher on the western side of the PRE and is mainly distributed in areas with numerous islands near the southern area of the PRE, basically in the same direction as the spread of freshwater from the Pearl River. This may be due to the alluvial accumulation of the mainland rivers, which brought a large amount of organic and inorganic substances, forming natural fishing grounds and spawning grounds near the islands [59]. From June to August, as a huge amount of Pearl River runoff enters the northern SCS, an alluvial freshwater hypersaline tongue forms between the PRE and the northern SCS and expands out to the open sea or the coast under the action of tides, monsoons and coastal currents [60]. The surface-diluted water extends to the eastern and western Guangdong waters in the form of a “quasi-symmetric extension” [4]. During this period, the major fishing grounds of larvae are influenced by alluvial freshwater and move out of the estuary, and their distribution characteristics are similar to the low saline tongue of alluvial freshwater. Preliminary investigations have shown that from June to August, under the influence of alluvial freshwater, the major fishing grounds of the PRE are alluvial to the vicinity of the Wanshan Islands and form one of the six fishing grounds in Guangdong, China, the Wanshan fishing ground [7], which are consistent with the predicted results of the model in this study. This is mainly due to the density of islands and reefs in this area, where freshwater and seawater currents meet and bring sufficient nutrients, producing a fishing ground for spawning, baiting and feeding [61,62,63,64]. In September, the southwest monsoon weakened and the GCC moved from northeast to southwest. Larvae were affected by the change in the current, and the high-value area of larval density moved to the west side of the PRE. At the same time, because the near-shore shallow waters were influenced by continental runoff and seawater masses, the salinity was relatively low and was rich in nutrients, providing ideal spawning grounds for fish and sufficient baits for the growth and development of larvae [64]. Therefore, larvae were mainly concentrated in the near-shore area on the west side of the PRE. In summary, the seasonal variation of larvae in the PRE was likely to be influenced by the combination of monsoon, Pearl River runoff, GCC and the invasion of high-salinity seawater from the outer sea.

## 5. Conclusions

In this study, the HSI model for the distribution of larvae in the PRE was constructed and the larvae habitat was predicted, based on satellite remote sensing and in situ observations. In June–August, the model constructed by MINM has the highest accuracy. In April and September, the model constructed by AMM and GMM has the highest accuracy. The interaction effects between environmental factors and the importance of the influencing factors will be considered, and the impact of daily environmental changes in the PRE on larvae will be discussed in future research, with a view to provide a more accurate scientific reference for the assessment and prediction of fishery resources in the PRE.

## Figures and Tables

**Table 1:** SI model for each environmental factor of larvae in the PRE.

Month	Variables	Single-Factor SI Model	*p*-Value
April	SST	SI = e^−1.353×(SST−23.197)^2^^	<0.001
	SSS	SI = e^−0.1238×(SSS−29.0204)^2^^	<0.001
	Chl-a	SI = e^−0.3079×(chla−2.9346)^2^^	<0.001
June	SST	SI = e^−16.20×(SST−29.64)^2^^	<0.001
	SSS	SI = e^−0.09026×(SSS−30.24479)^2^^	<0.001
	Chl-a	SI = e^−0.154×(Chla−1.878)^2^^	<0.001
July	SST	SI = e^−18.63×(SST−28.96)^2^^	<0.001
	SSS	SI = e^−0.06309×(SSS−27.90948)^2^^	<0.001
	Chl-a	SI = e^−0.07337×(Chla−4.09307)^2^^	<0.001
August	SST	SI = e^−11.63×(SST−29.20)^2^^	<0.001
	SSS	SI = e^−0.04606×(SSS−29.60043)^2^^	<0.001
	Chl-a	SI = e^−0.121×(Chla−3.673)^2^^	<0.001
September	SST	SI = e^−13.02×(SST−28.68)^2^^	<0.001
	SSS	SI = e^−0.08401×(SSS−24.42966)^2^^	<0.001
	Chl-a	SI = e^−0.2262×(Chla−2.3911)^2^^	<0.001

**Table 2:** The optimum value of environmental factors for larvae in the PRE.

Month	SST (°C)	SSS	Chl-a (mg/m^3^)
April	23.2 ± 0.4	29.0 ± 1.3	2.9 ± 0.8
June	29.6 ± 0.1	30.2 ± 1.5	1.9 ± 1.2
July	29.0 ± 0.1	27.9 ± 1.9	4.1 ± 1.7
August	29.2 ± 0.1	29.6 ± 2.1	3.7 ± 1.4
September	28.7 ± 0.1	24.4 ± 1.6	2.4 ± 1.0

**Table 3:** Accuracy validation of HSI models.

Month	Arithmetic Mean Model (AMM)	Geometric Mean Model (GMM)	Maximum Model (MAXM)	Minimum Model (MINM)
April	71%	71%	57%	57%
June	58%	64%	41%	70%
July	61%	76%	46%	84%
August	36%	36%	36%	64%
September	93%	93%	60%	87%

## Data Availability

The data presented in this study are available on request from the corresponding author. The data are not publicly available due to privacy policy.
